# Supplementary material for: Nucleolin Targeting by N6L Inhibits Wnt/β-Catenin Pathway Activation in Pancreatic Ductal Adenocarcinoma
Source: Cancers (Basel). 2021 Jun 15;13(12):2986. doi: 10.3390/cancers13122986 (PMC8232280; doi:10.3390/cancers13122986)

Figure 1C mPDAC - pull-down – anti-β-catenin (duplicate)

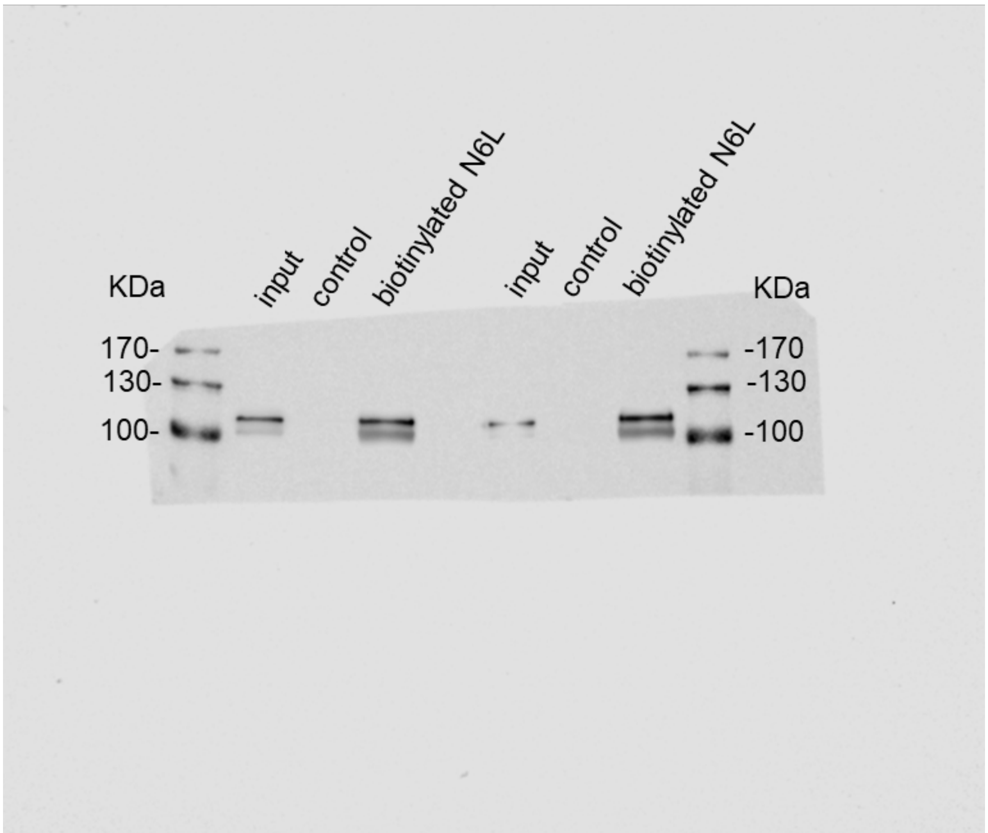

Figure 1B mPDAC – pull-down – anti-nucleolin (duplicate)

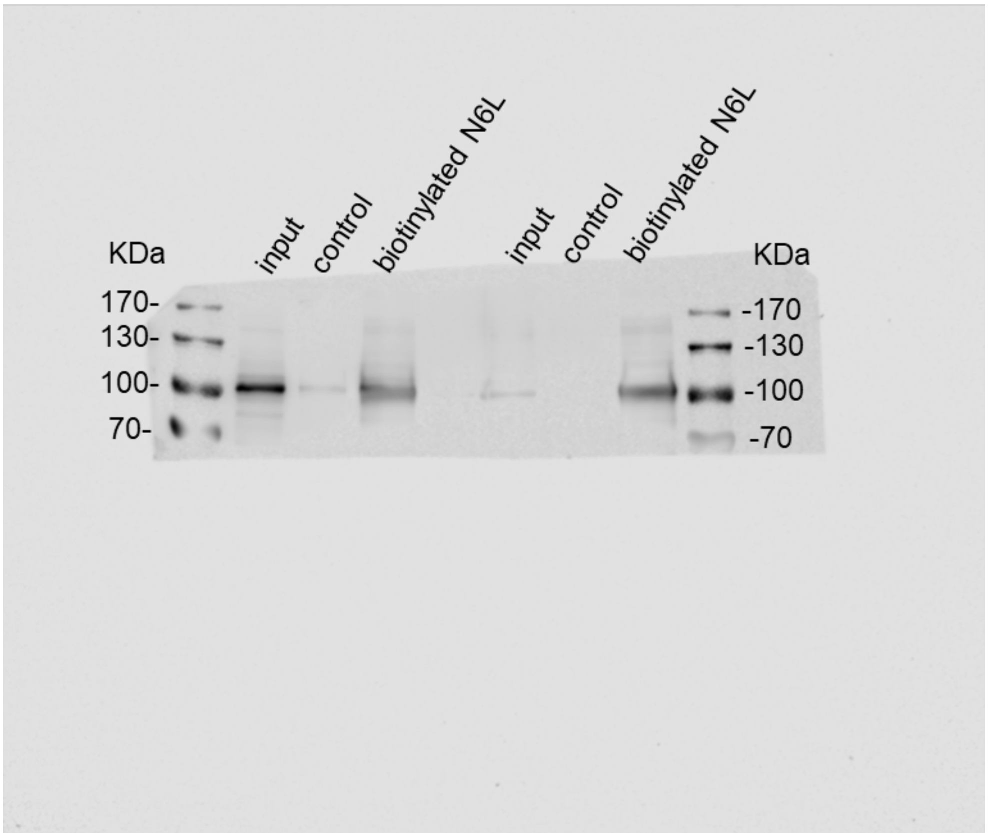

Figure 1C MIA PaCa-2 – pull-down – anti-β-catenin (duplicate)

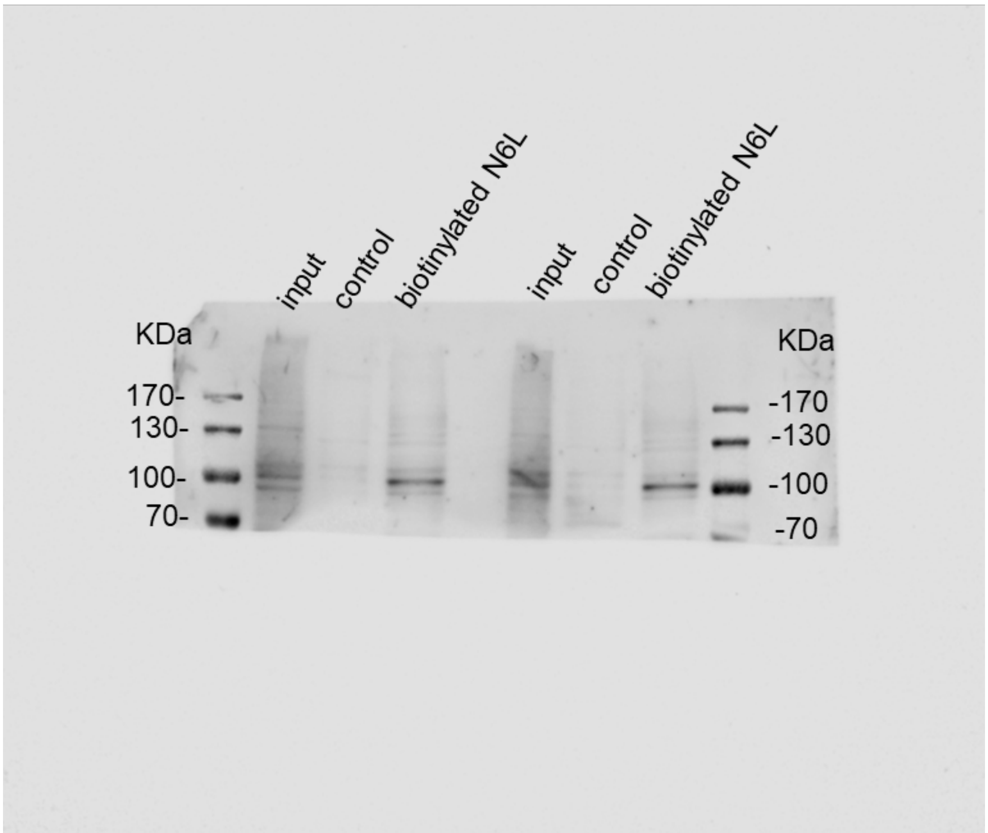

Figure 1C MIA PaCa-2 – pull-down – anti-nucleolin (duplicate)

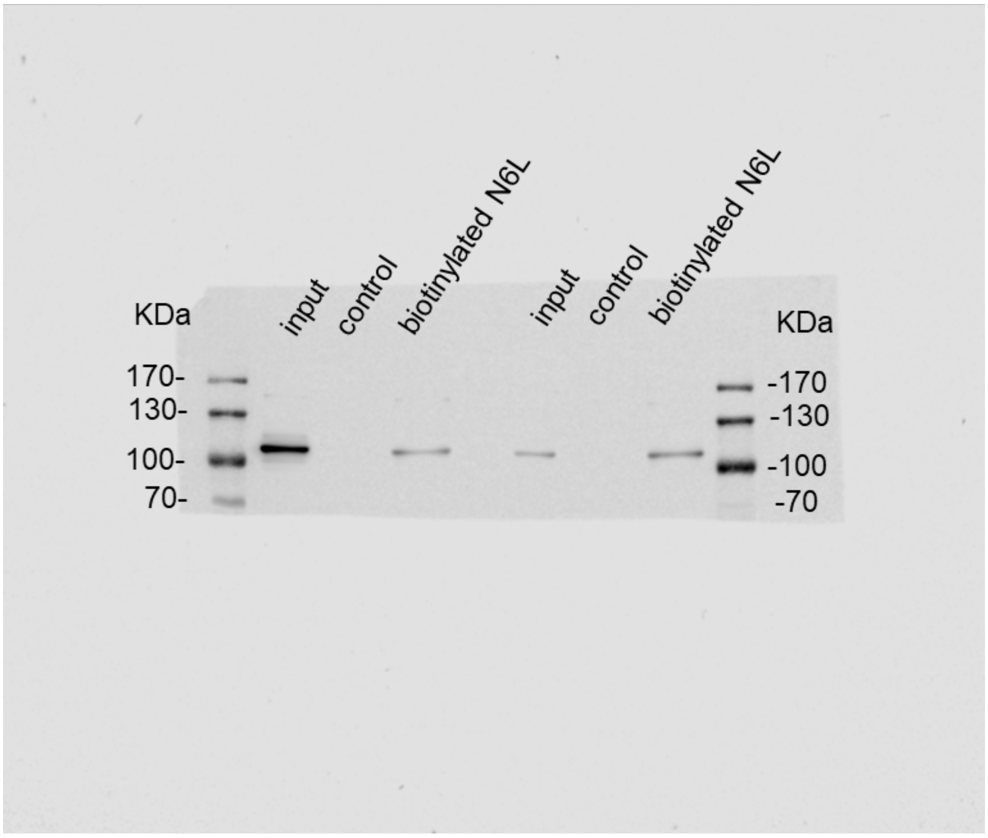

Figure 2D MIA PaCa-2 – N6L+Wnt3A – anti-cyclin D1

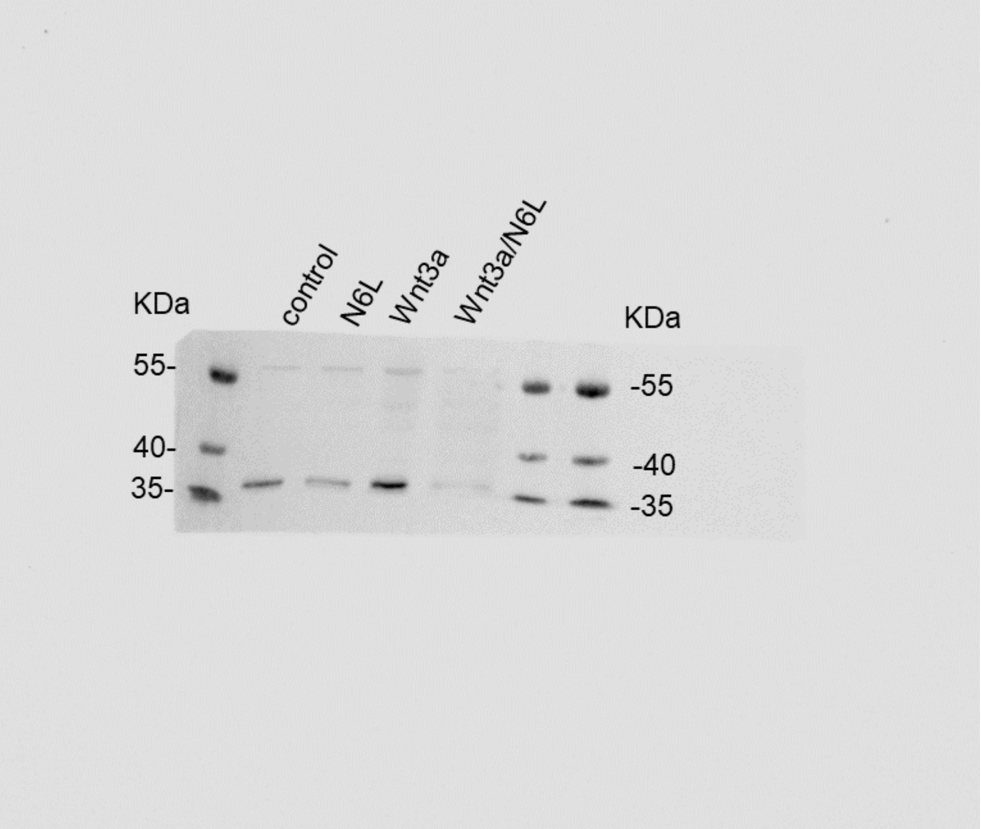

Figure 2D MIA PaCa-2 – N6L+Wnt3A – anti-β-actin

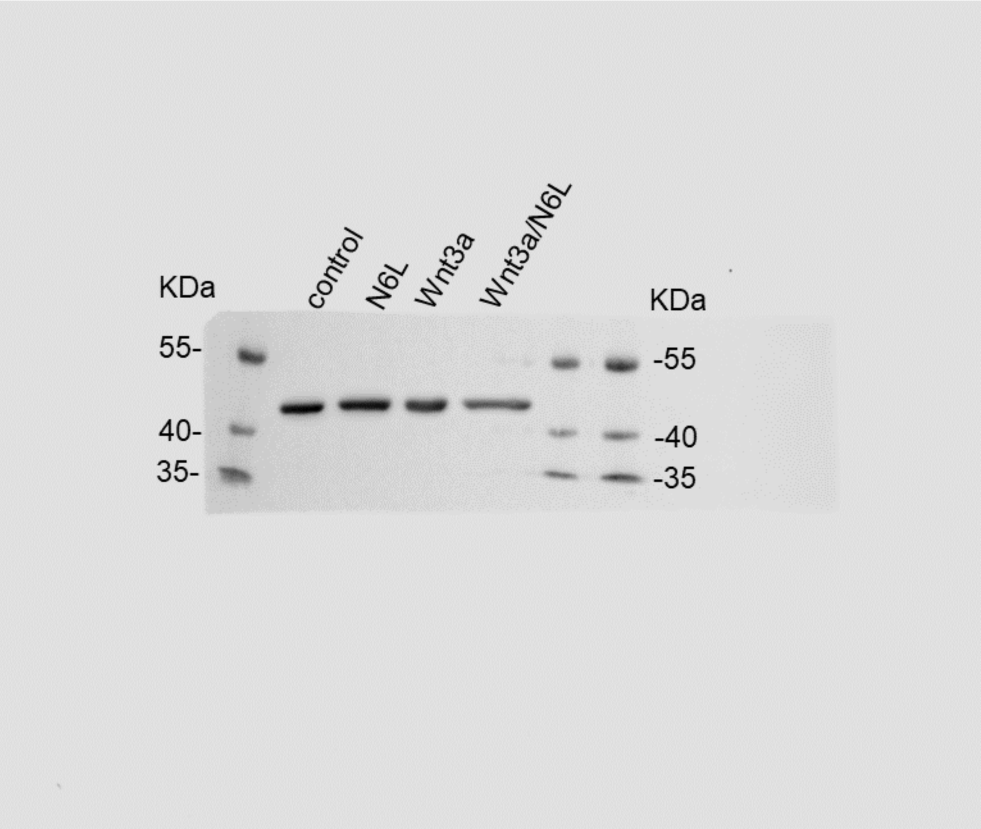

Figure 2F MIA PaCa-2 – N6L+Wnt3A – anti-β-catenin

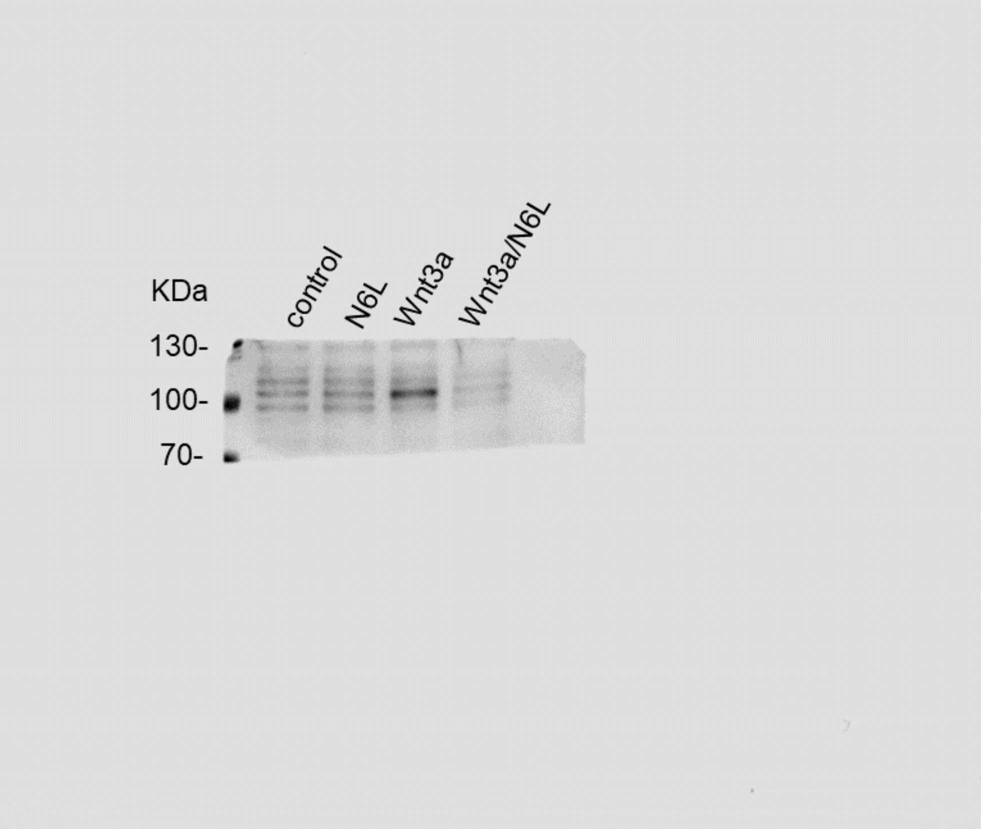

Figure 2F MIA PaCa-2 – N6L+Wnt3A – anti-active β-catenin

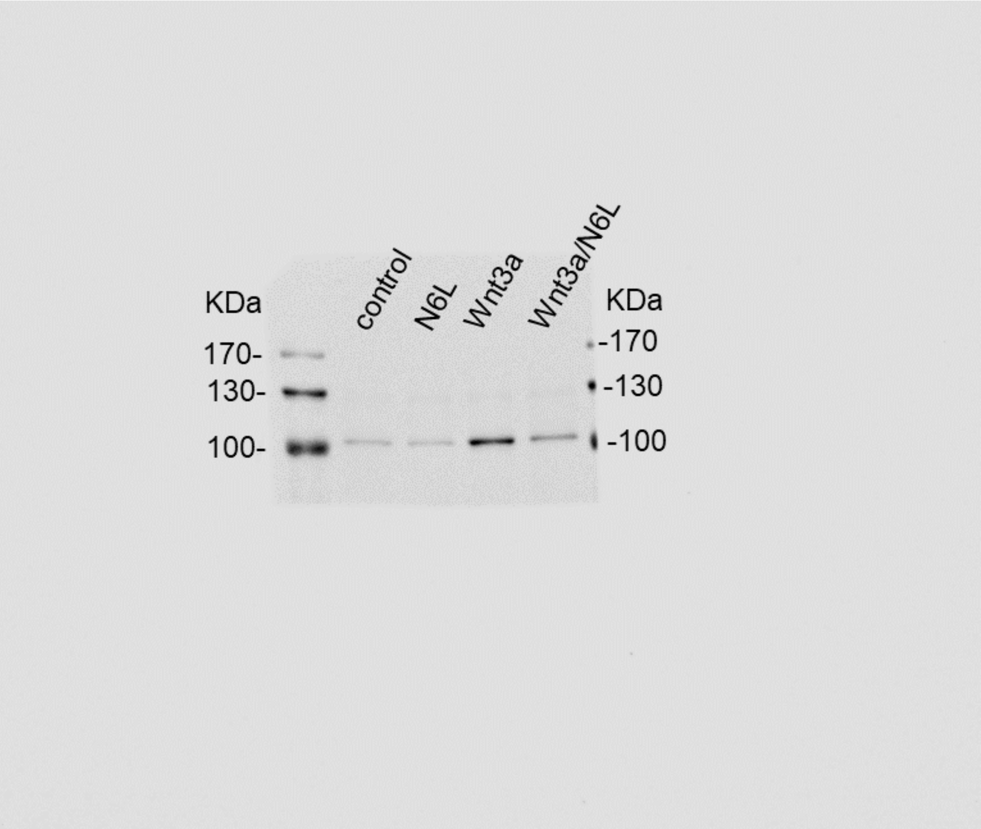

Figure 2F MIA PaCa-2 – N6L+Wnt3A – anti-β-actin

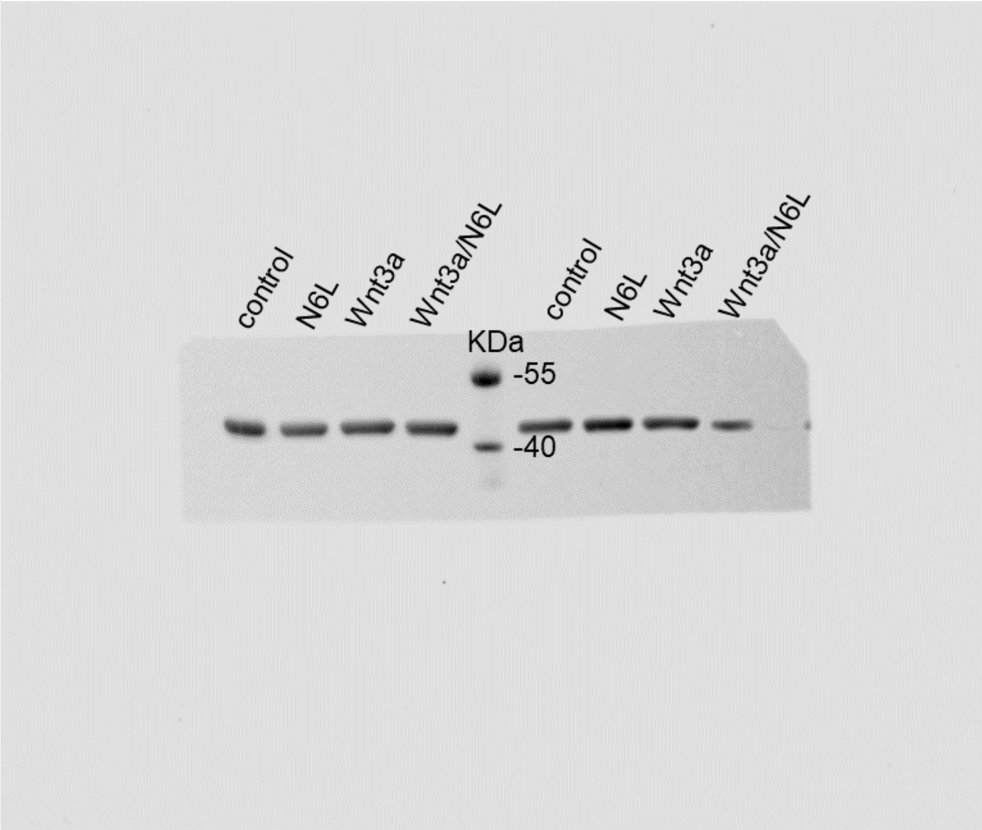

Figure 2I MIA PaCa-2 – N6L+TWS119 – anti-active β-catenin

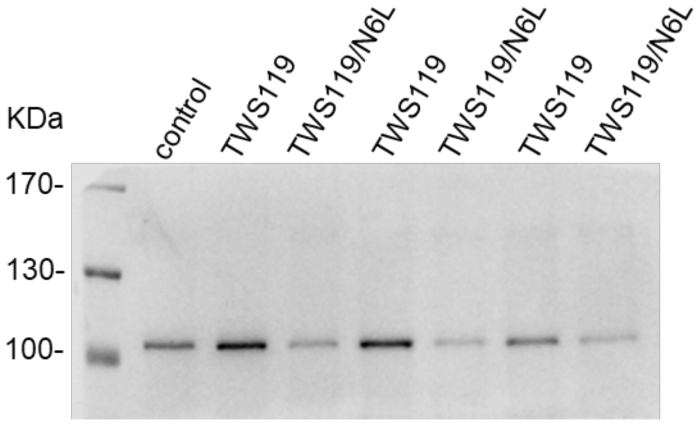

Figure 2I MIA PaCa-2 – N6L+TWS119 – anti-β-actin

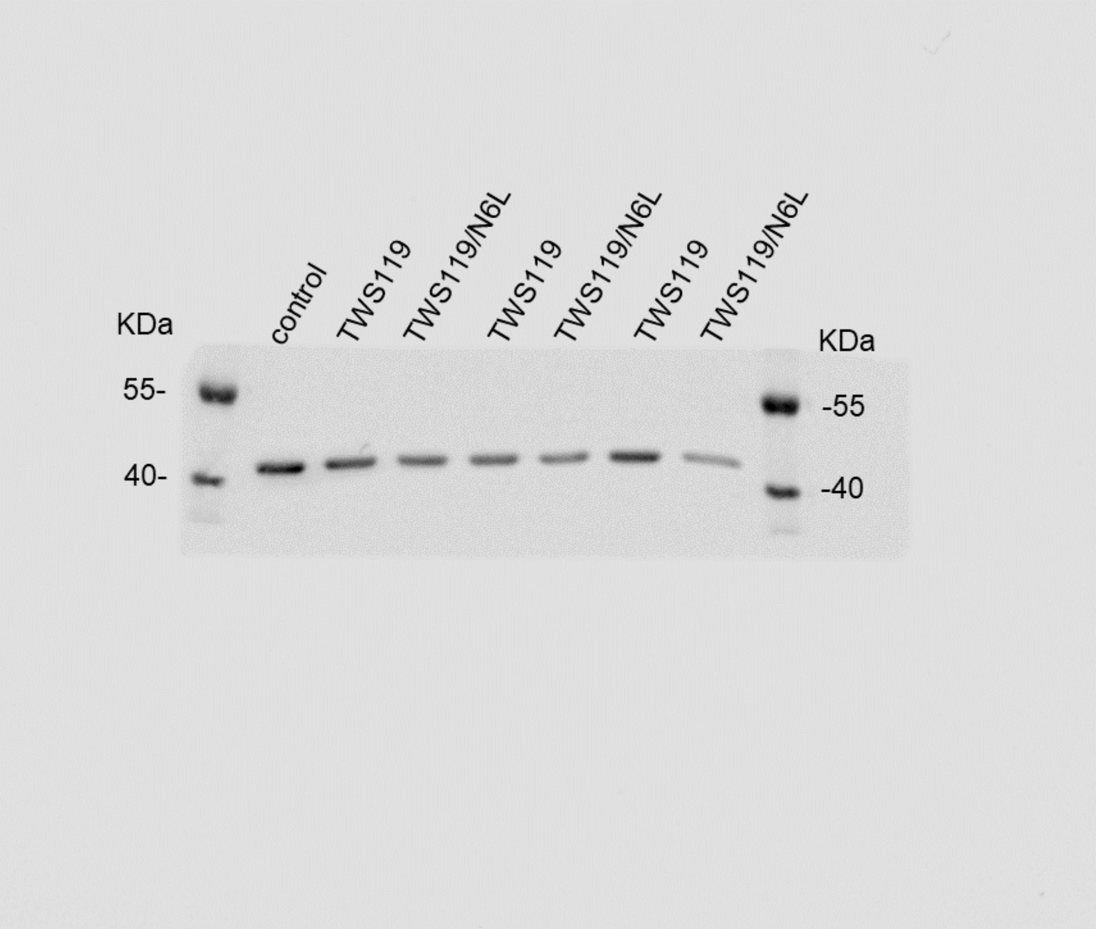

Figure 3A MIA PaCa-2 – Nucleolin IP – anti-β-catenin

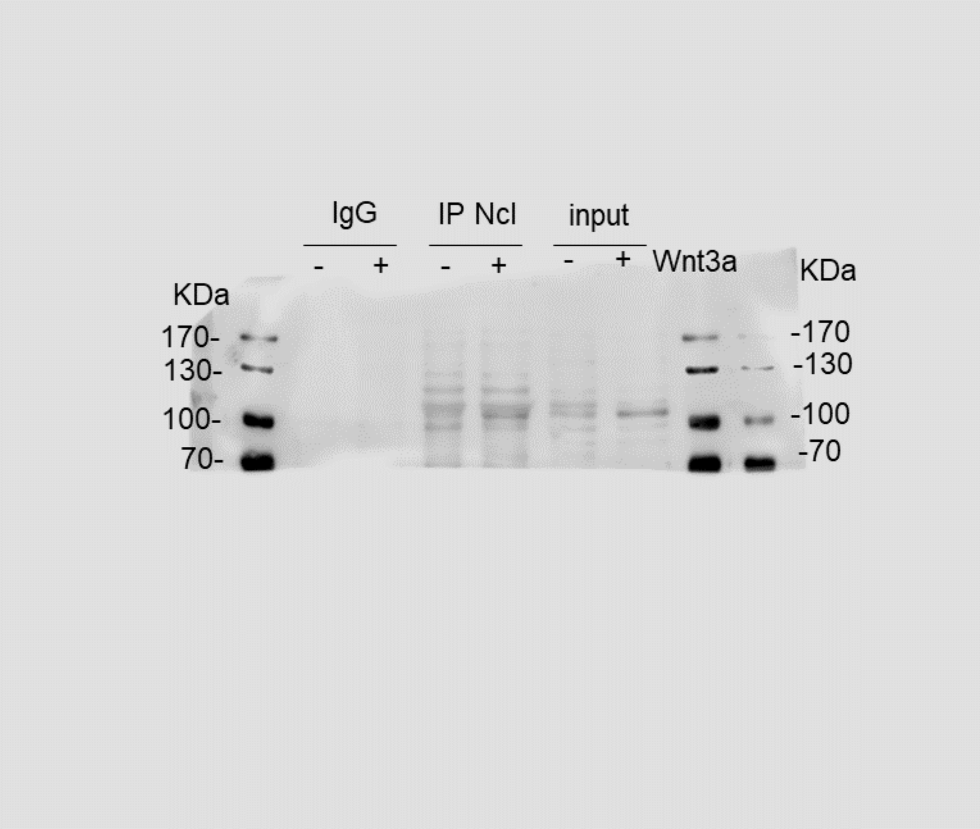

Figure 3A MIA PaCa-2 – Nucleolin IP – anti-nucleolin

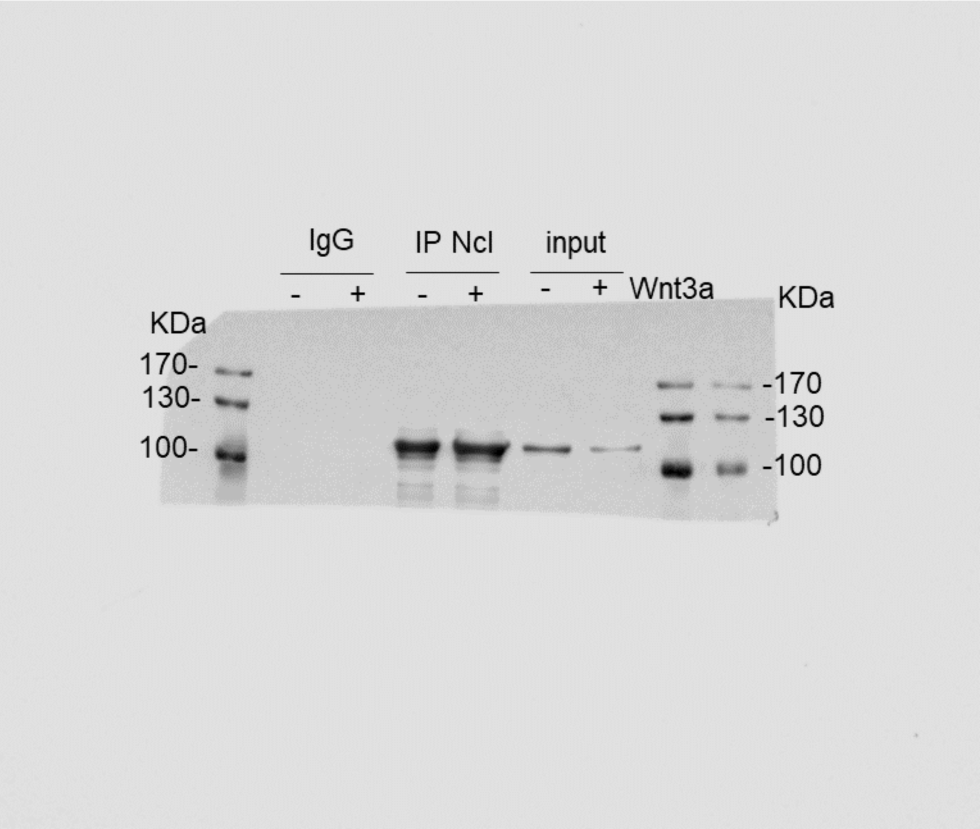

Figure 3B MIA PaCa-2 – siNCL+Wnt3A – anti-active β-catenin

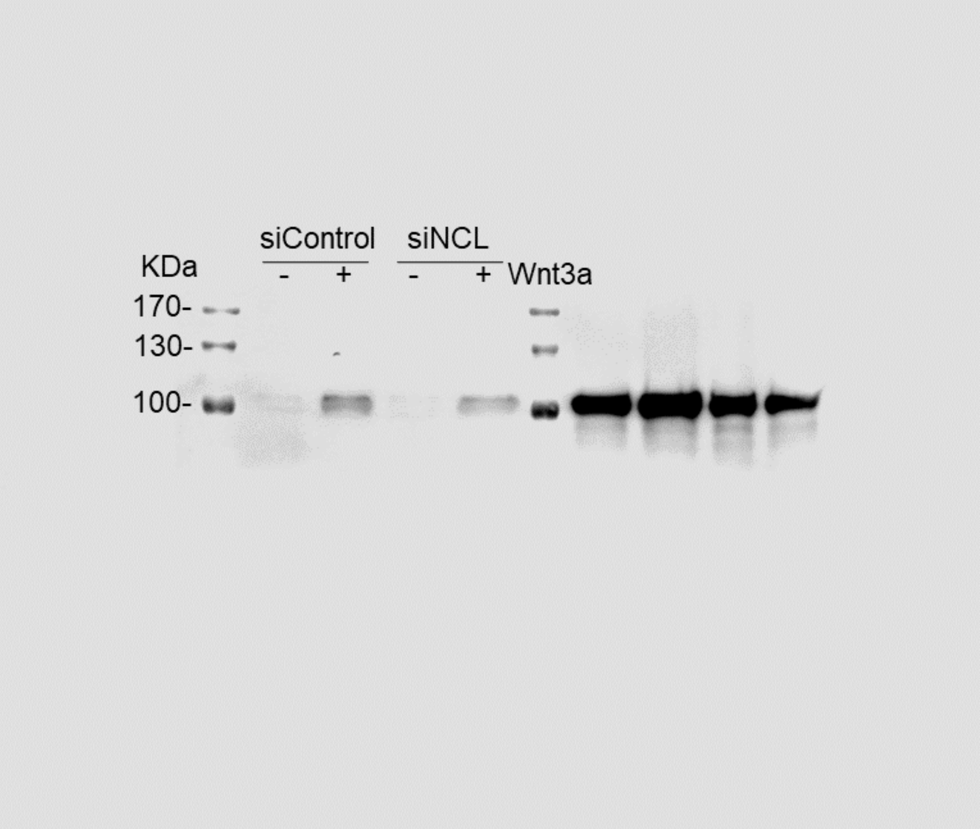

Figure 3B MIA PaCa-2 – siNCL+Wnt3A – anti-nucleolin

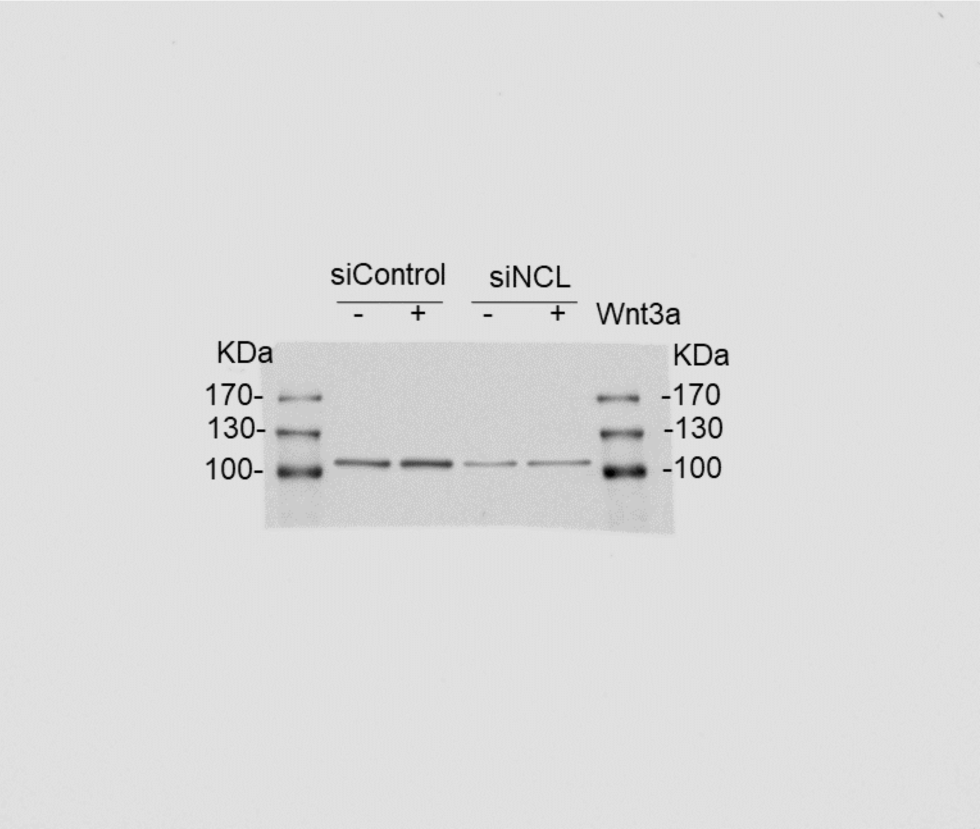

Figure 3B MIA PaCa-2 – siNCL+Wnt3A – anti-β-actin

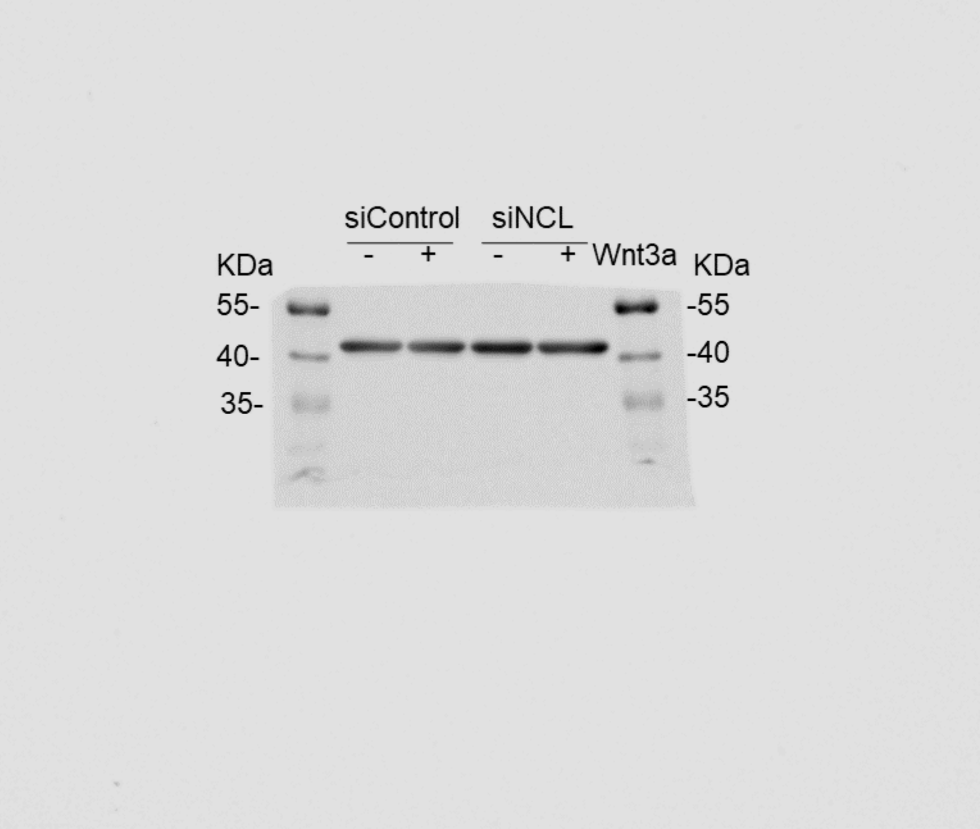

Figure 3D MIA PaCa-2 – NCL-GFP+Wnt3A – anti-GFP

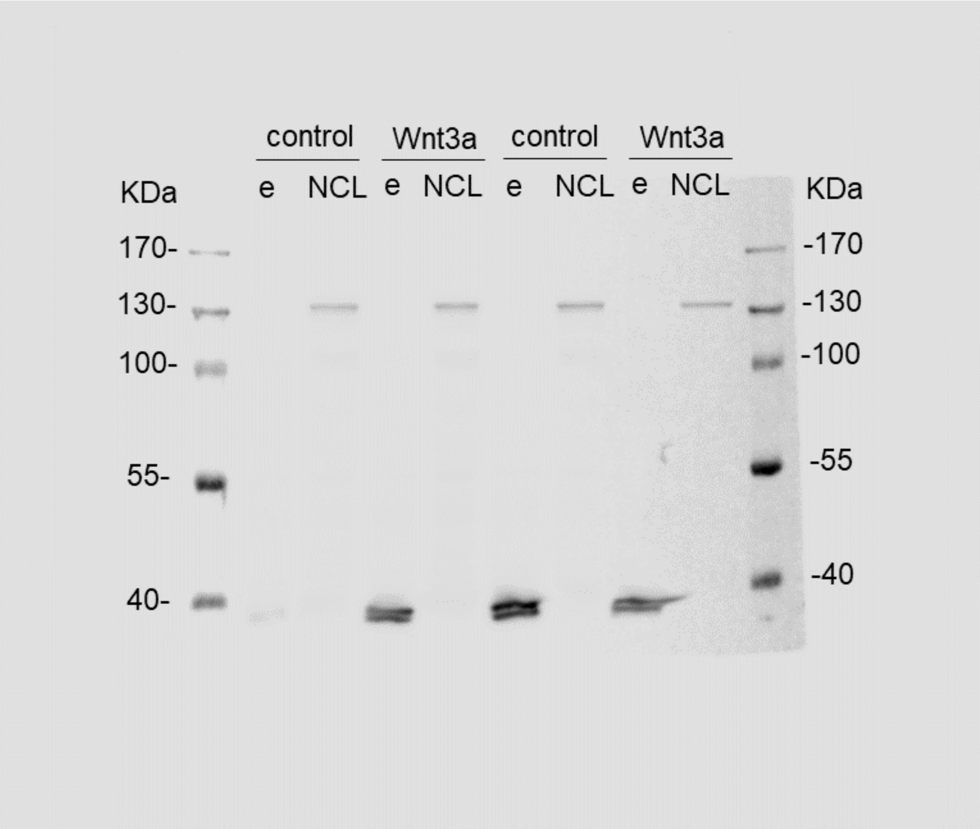

Figure 3D MIA PaCa-2 – NCL-GFP +Wnt3A – anti-active β-catenin and anti-nucleolin

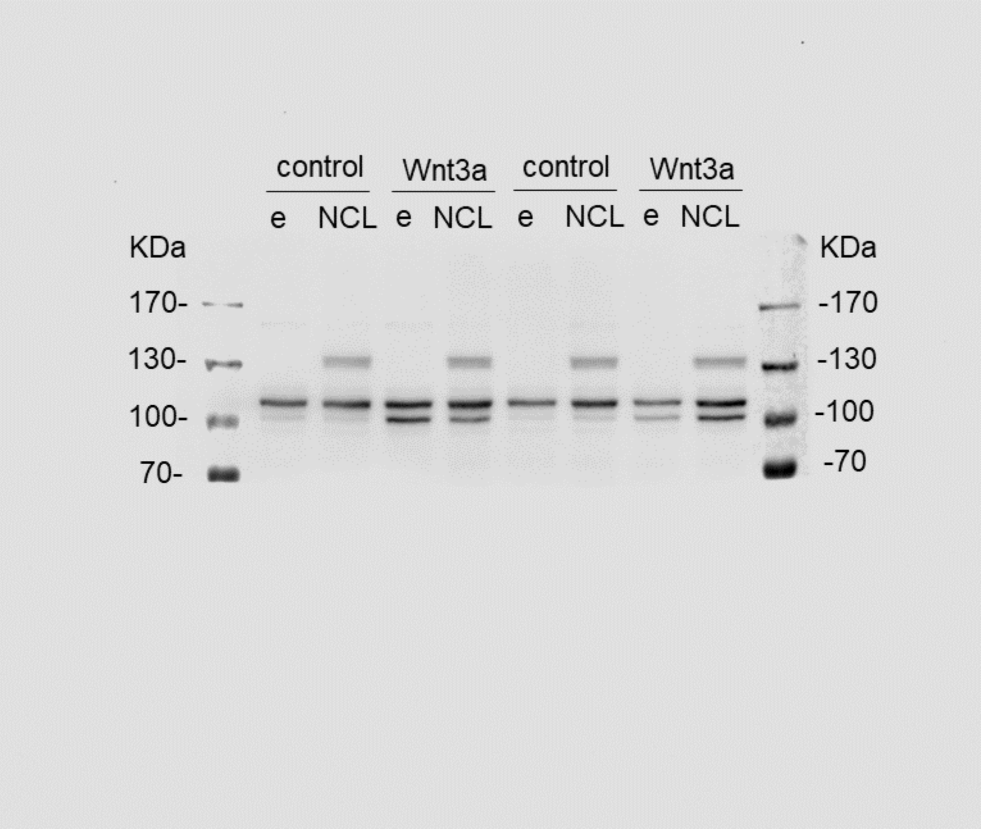

Figure 3D MIA PaCa-2 – NCL-GFP +Wnt3A – anti-β-actin

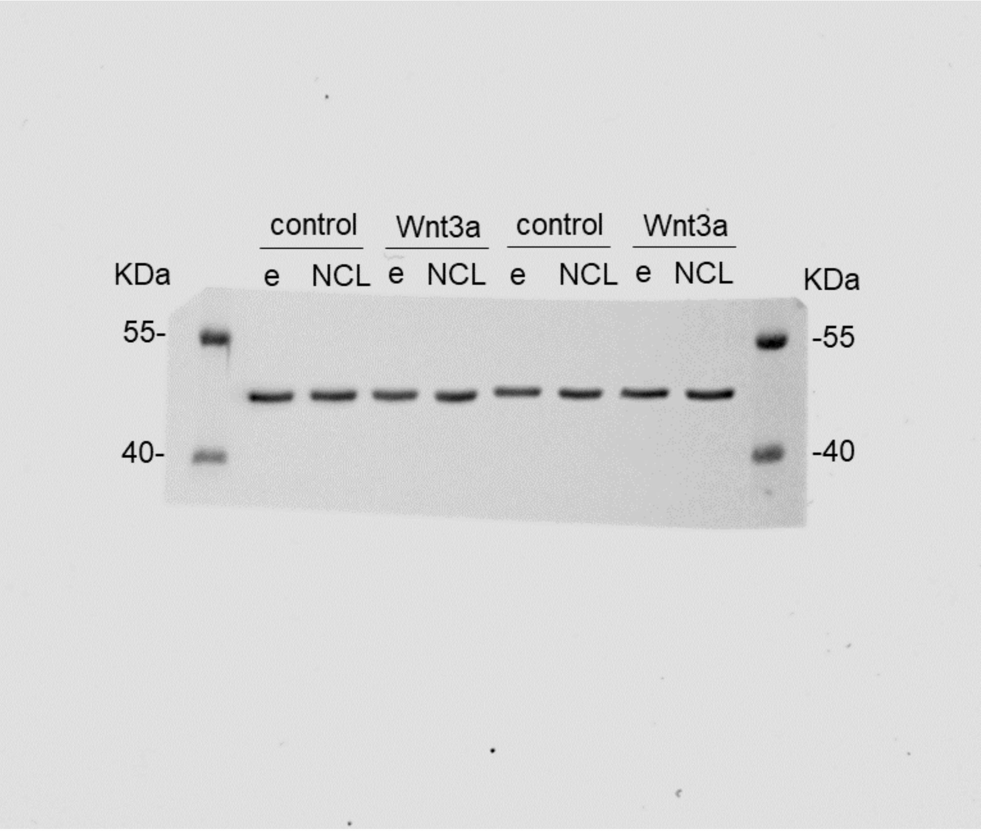

Supplement: Supplementary file 1 [file cancers-13-02986-s001.zip › cancers-1183366-supplementary/cancers-1183366-original western blotting figures.pdf]
