# Supplementary material for: Nucleolin Targeting by N6L Inhibits Wnt/β-Catenin Pathway Activation in Pancreatic Ductal Adenocarcinoma
Source: Cancers (Basel). 2021 Jun 15;13(12):2986. doi: 10.3390/cancers13122986 (PMC8232280; doi:10.3390/cancers13122986)
Supplement: Supplementary file 1 [file cancers-13-02986-s001.zip › cancers-1183366-supplementary/cancers-1183366-supplementary.pdf]

cancers

MDPI

Supplementary materials and methods

Nucleolin Targeting by N6L Inhibits Wnt/β-Catenin Pathway Activation in Pancreatic Ductal Adenocarcinoma

Fabio Raineri, Sandrine Bourgoin-Voillard, Mélissande Cossutta, Damien Habert, Matteo Ponzo [1], Claire Houppe, Benoît Vallée, Michele Boniotto, Mounira Chalabi-Dchar, Philippe Bouvet, Anne Couvelard, Jerome Cros, Anais Debesset, José L. Cohen, José Courty and Ilaria Cascone

1.1 Antibodies

For Western Blotting analysis, the following primary antibodies were used: anti-nucleolin (Abcam ab22758, 1:1000), anti-β-catenin (Sigma C2206, 1:1000), anti-non-phospho (active) β-catenin (Cell Signaling #8814, 1:1000), anti-GFP (Santa Cruz sc-9996, 1:200), anti-Cyclin D1 (Cell signaling #2978, 1:1000), anti-phospho-Ser9-GSK3β (XX, 1:500) and anti-β-actin HRP (Sigma A-5441, 1:10000). Anti-rabbit IgG HRP (Jackson 111-035-144, 1:10000) and anti-mouse IgG HRP antibodies (Jackson 705-065-147, 1:10000) were used as secondary antibodies. For immunofluorescence, the following primary antibodies were used: anti-non-phospho (active) β-catenin (Cell Signaling #8814, 1:800) and anti-Cyclin D1 (Cell signaling #2978, 1:800). Anti-rabbit IgG Alexa Fluor 488 antibody (Thermo Scientific A-21206, 1:400) was used as secondary antibody.

1.2. Pull-down with biotinylated-N6L

Synthesis of N6L is carried out as previously described (Destouches et al., 2011). Biotinylated-N6L was prepared by inducing a disulfide bond between biotin and Cys-N6L (Destouches et al., 2011). Increasing concentrations of biotinylated-N6L were incubated on mPDAC and MIA PaCa-2 cells at room temperature for 45 minutes. A subcellular extra-nuclear lysis was performed as described in (Callebaut et al., 1996) to harvest membrane and cytoplasmic proteins while excluding nuclear proteins. The affinity-purification assay based on streptavidin sepharose beads (GE Healthcare, Life Sciences, 17-5113-01) and biotinylated-N6L was performed on 1 mg of protein extract. Biotin alone was used as negative control. Protein complexes were eluted, separated by 8% SDS-PAGE and colored by Commassie blue.

1.3. Proteomic analysis

Each band of the SDS-PAGE of biotinylated-N6L pull-down was submitted to in-gel digestion by using Trypsin 3% (Sigma) after performing a reduction of disulfide bonds with DTT and cysteine alkylation with iodoacetamide. Resulting peptides were extracted and analyzed on a nanoLC-C18/MS system, a nanoAcquity UPLC (Waters) coupled to a SYNAPT G2-Si High Definition Mass Spectrometer (Waters) equipped with a nano-electrospray ionization source operating in a positive mode. In the nanoAcquity UPLC system, samples were desalted through a C18 trapping column (NanoACQUITY Symmetry C18, 20mm, 180μm, 100Å, 5μm, Waters) before separating peptides according to their hydrophobicity on a C18 reverse phase nano-column (nanoACQUITY peptide BEH C18, 100 mm, 100μm, 130Å, 1.7μm, 100μm, Waters). The SYNAPT G2-Si High Definition mass spectrometer was operated in MSE mode, a mode in which the MSE technology uses the high-efficiency T-Wave ion mobility cell to fractionate ions according to their charge states/shapes, and improve sensitivity and

specificity of the analysis. Identification of peptides and resulting proteins was performed by analyzing MS and MSE data with PLGS software (Protein Lynx Global Server, version 3.1, Waters) and the UniprotKB/Swissprot database (Release-2019_02) and by considering: carboamidomethylation as a fixed modification, oxidation of methionine as a variable modification, 1 missed cleavage allowed, lock mass for charge 3 of ionized leucine enkephalin (m/z 556.2771 ± 0.25 u), mass precursor tolerance: automatic and fragment ion tolerance: automatic. Only proteins matching with the following criteria were retained as identified: a PLGS confident score equal to 2, a minimum of 2 unique peptides (Supplementary Table 1) for a FDR (False Discovery Rate) of 4%. Non-specific proteins found in the negative control (biotinylated control) were subtracted from the list of the specific proteins interacting with N6L. This complete analysis was performed in duplicates. Proteins exclusively identified in N6L pull-down samples for both duplicates were retained as N6L partners. A network of these N6L protein partners in PDAC (N6L interactome in Supplementary Figure 2) was built by using Cytoscape software (version 3.8.0) (Shannon et al., 2003) with StringApp v1.5.1 and STRING database (Doncheva et al., 2019). Gene ontology of this network was obtained by using Cytoscape software with ClueGO (version 2.5.6) / CluePedia (version 1.5.6) applications (Bindea et al., 2013, 2009). WikiPathway (Kutmon et al., 2016) was used to build the Wnt pathway into Cytoscape and to establish the connection of Wnt pathway with N6L network.

1.4. TMA staining and analysis

An immunohistochemical staining was performed with anti-active β-catenin antibody using standard protocol in 45 human PDAC included in a tissue microarray (TMA) paraffin block. Immunostaining, was performed using an automatized technique (Streptavidin-peroxydase with an automate Bond Max, Leica), and slides were counterstained with hematoxylin. Images were taken by the by Scanner Aperio Scanscope CS. Analysis of active β-catenin staining was performed by a score determination corresponding to the intensity of the labeling of tumor cells from 0 to 3 (0, no staining; 1, low staining; 2, moderate staining; 3, high staining) in each spot. The final score for each tumor was the average of the scores obtained for each spot available by tumor.

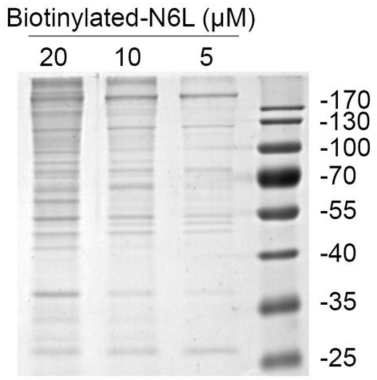

Supplementary Figure S1.

Colloidal Blue gel showing the protein bands from 5, 10 and 20 μM biotinylated-N6L pull-down. The protein band intensity increased by increasing the biotinylated-N6L concentration.

Figure S1: Biotinylated-N6L pull-down.

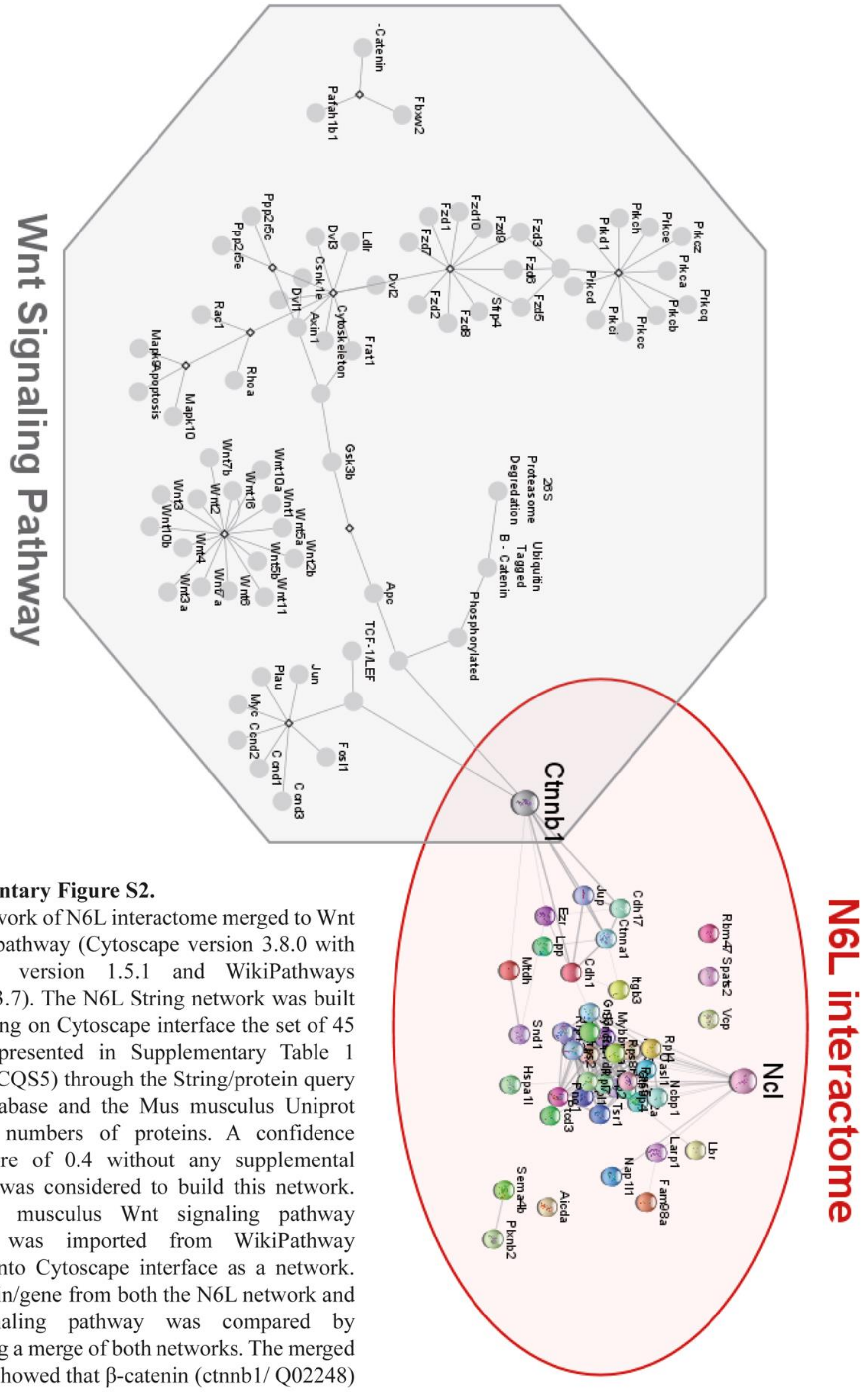

Supplementary Figure S2.
String network of N6L interactome merged to Wnt signaling pathway (Cytoscape version 3.8.0 with StringApp version 1.5.1 and WikiPathways version 3.3.7). The N6L String network was built by importing on Cytoscape interface the set of 45 proteins (presented in Supplementary Table 1 except Q9CQS5) through the String/protein query public database and the Mus musculus Uniprot accession numbers of proteins. A confidence cutoff score of 0.4 without any supplemental interactor was considered to build this network. The Mus musculus Wnt signaling pathway (WP403) was imported from WikiPathway database into Cytoscape interface as a network. This protein/gene from both the N6L network and Wnt signaling pathway was compared by considering a merge of both networks. The merged networks showed that β-catenin (ctnnb1/ Q02248) interplays in both N6L and Wnt signaling pathway networks.

Figure S2: N6L interactome.

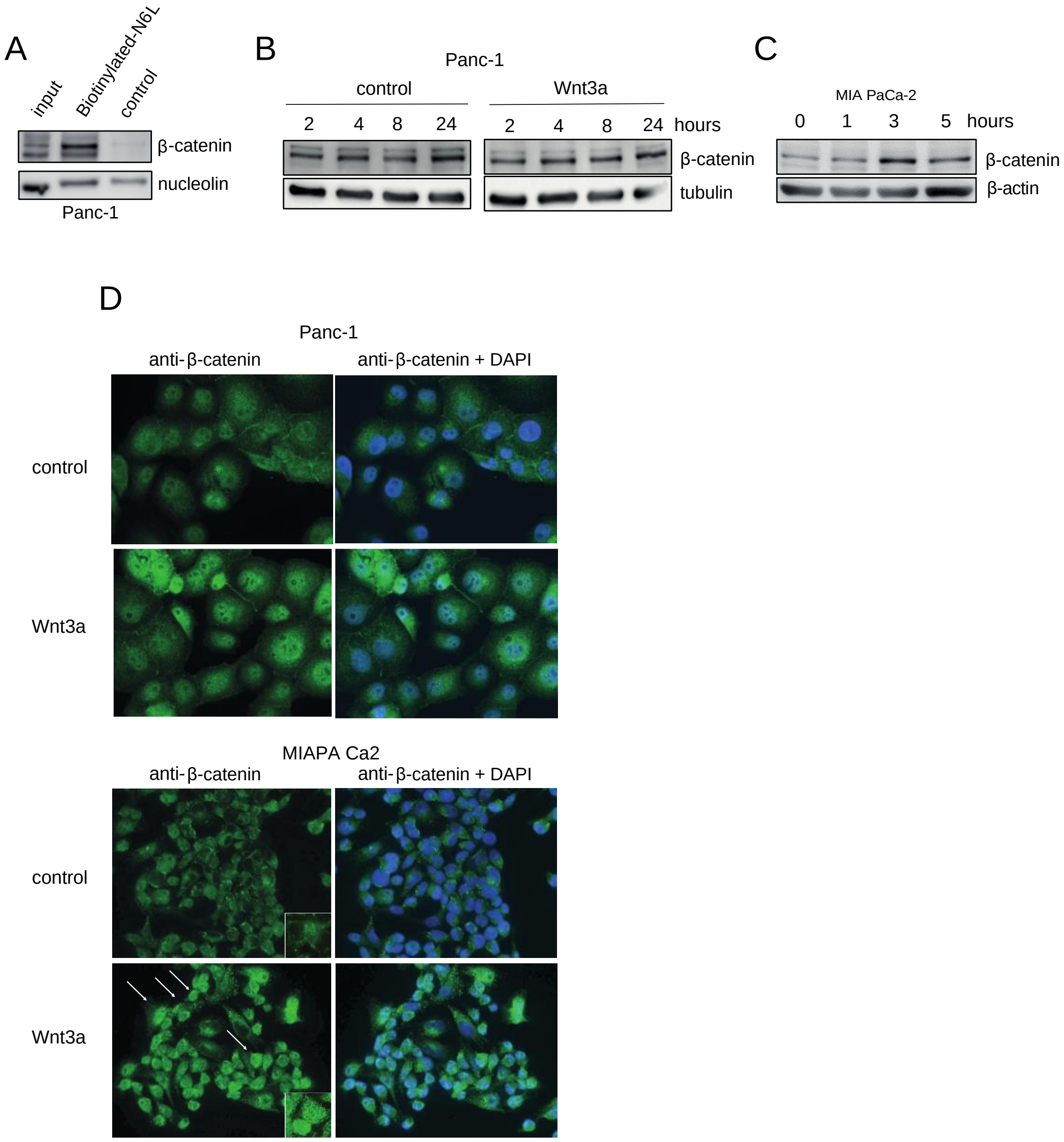

Supplementary Figure S3. MIA PaCa-2 and Panc-1 cell comparison.
(**A**) The cell lysate of the extranuclear cellular fraction (input) and the biotinylated N6L pull-down of Panc-1 cells were analyzed by Western Blotting with anti-nucleolin and b-catenin antibodies. (**B** and **C**) Western Blotting of active and total b-catenin of MIA PaCa-2 and Panc-1 cells stimulated by Wnt3a-CM at the indicated different time points. (**D**) Immunofluorescence staining of b-catenin in Panc-1 (upper) and MIA PaCa-2 (down) cells stimulated or not by Wnt3A by using an anti-b-catenin antibody. Nuceli were stained by DAPI. In MIA PaCa2 cells, b-catenin localizes to the nuclei after stimulation with Wnt3A (arrows) wile in Panc-1 cells, b-catenin was already present in the nuclei of unstimulated cells

Figure S3. MIAPA Ca2 and Panc-1 cell comparison.

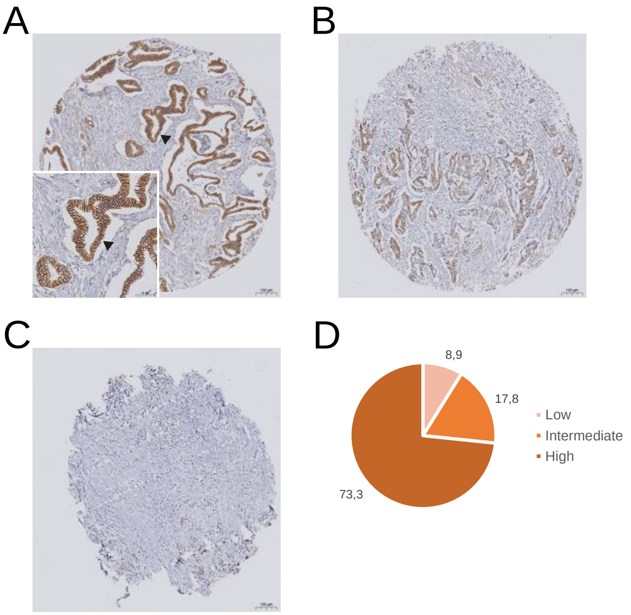

Supplementary Figure S4.
Human PDAC included in a TMA was immunostained with an anti-non-phosphorylated β-catenin antibody and scored as high **(A)**, intermediate **(B)**, low **(C)** or negative (see Materials and Methods). Active β-catenin was highly expressed by tumoral ducts (arrowhead in **A**).

Figure S4. Active β-catenin staning in PDAC patients.

Table S1: Mass-spectometry analysis of N6L pull-down proteins.

Uniprot Accession Number	Entry	Protein name	PLGS Score	Peptides	Coverage (%)	nucleolin interaction
P09405	NUCL_MOUSE	Nucleolin	4870	60	46	
O54890	ITB3_MOUSE	Integrin beta-3	573	18	26	(Koutsioumpa et al., 2013)
P12970	RL7A_MOUSE	60S ribosomal protein L7A	11638	38	64	(Salvetti et al., 2016)
P14148	RL7_MOUSE	60S ribosomal protein L7	16104	52	59	(Salvetti et al., 2016)
P35980	RL18_MOUSE	60S ribosomal protein L18	7796	15	39	(Salvetti et al., 2016)
P47962	RL5_MOUSE	60S ribosomal protein L5	11788	32	48	(Salvetti et al., 2016)
P62242	RS8_MOUSE	40S ribosomal protein S8	15051	27	64	(Salvetti et al., 2016)
P97461	RS5_MOUSE	40S ribosomal protein S5	6322	15	40	(Salvetti et al., 2016)
Q14C51	PTCD3_MOUSE	Pentatricopeptide repeat domain-containing protein 3_ mitochondrial	471	29	29	(Salvetti et al., 2016)
Q6ZQ58	LARP1_MOUSE	La-related protein 1	1742	47	38	(Burrows et al., 2010)
Q78PY7	SND1_MOUSE	Staphylococcal nuclease domain-containing protein 1	3679	60	40	(Cappellari et al., 2013)
Q7TMK9	HNRPQ_MOUSE	Heterogeneous nuclear ribonucleoprotein Q	1941	35	32	(Peddigari et al., 2013)
Q7TPV4	MBB1A_MOUSE	Myb-binding protein 1A	745	32	24	(Mori et al., 2012)
Q8BVY0	RL1D1_MOUSE	Ribosomal L1 domain-containing protein 1	424	13	24	(Salvetti et al., 2016)
Q8CI11	GNL3_MOUSE	Guanine nucleotide-binding protein-like 3	417	14	31	(Salvetti et al., 2016)
Q8VEK3	HNRPU_MOUSE	Heterogeneous nuclear ribonucleoprotein U	865	24	25	(Salvetti et al., 2016)
Q922K7	NOP2_MOUSE	Probable 28S rRNA (cytosine-C(5))-methyltransferase	177	15	24	(Salvetti et al., 2016)
Q99ME9	NOG1_MOUSE	Nucleolar GTP-binding protein 1	1218	18	25	(Salvetti et al., 2016)
Q9D8E6	RL4_MOUSE	60S ribosomal protein L4	7539	52	47	(Shen et al., 2016)
B2RXS4	PLXB2_MOUSE	Plexin-B2	2607	88	36	
P09803	CADH1_MOUSE	Cadherin-1	2874	50	23	
P16627	HS71L_MOUSE	Heat shock 70 kDa protein 1-like	2034	19	33	
P26040	EZRI_MOUSE	Ezrin	230	22	32	
P26231	CTNA1_MOUSE	Catenin alpha-1	18797	115	62	

P28656	NP1L1_MOUSE	Nucleosome assembly protein 1-like 1	7106	17	26	
Q01853	TERA_MOUSE	Transitional endoplasmic reticulum ATPase	1060	31	37	
Q02248	CTNB1_MOUSE	Catenin beta-1	6898	59	39	
Q02257	PLAK_MOUSE	Junction plakoglobin	5642	51	57	
Q3TJZ6	FA98A_MOUSE	Protein FAM98A	332	13	32	
Q3U9G9	LBR_MOUSE	Lamin-B receptor	494	14	16	
Q3UYV9	NCBP1_MOUSE	Nuclear cap-binding protein subunit 1	821	14	15	
Q5SWD9	TSR1_MOUSE	Pre-rRNA-processing protein TSR1 homolog	1665	36	26	
Q62179	SEM4B_MOUSE	Semaphorin-4B	744	18	21	
Q6P5B0	RRP12_MOUSE	RRP12-like protein	1002	46	30	
Q80WJ7	LYRIC_MOUSE	Protein LYRIC	1834	23	35	
Q8BFW7	LPP_MOUSE	Lipoma-preferred partner homolog	266	12	28	
Q8BJW6	EIF2A_MOUSE	Eukaryotic translation initiation factor 2A	1751	26	43	
Q8K1N4	SPAS2_MOUSE	Spermatogenesis-associated serine-rich protein 2	325	15	37	
Q8VI94	OASL1_MOUSE	2'-5'-oligoadenylate synthase-like protein 1	2463	20	44	
Q91WT8	RBM47_MOUSE	RNA-binding protein 47	317	14	24	
Q924T2	RT02_MOUSE	28S ribosomal protein S2, mitochondrial	243	3	13	
Q9CPS7	PNO1_MOUSE	RNA-binding protein PNO1	2006	11	42	
Q9CQS5	RIOK2_MOUSE	Serine/threonine-protein kinase RIO2	433	15	22	
Q9R100	CAD17_MOUSE	Cadherin-17	830	16	24	
Q9WVE0	AICDA_MOUSE	Single-stranded DNA cyt	190	7	29	

Supplementary Table 1. Proteins identified by mass-spectrometry from N6L pull-down in mPDAC cells. Group of 45 proteins found by performing the biotinylated-N6L pull-down of mPDAC cells. 18 proteins are referenced to interact with nucleolin.

Table S2: Gene onthology (GO) analysis of N6L pull-down proteins.

GOID	GOTerm	Ontology Source	Term PValue
GO:000644	regulation of translational initiation	GO_BiologicalProcess-EBI-UniProt-GOA_10.12.2019_00h00	3,98E-04
GO:004225	ribosome assembly	GO_BiologicalProcess-EBI-UniProt-GOA_10.12.2019_00h00	1,13E-05
GO:004227	ribosomal small subunit biogenesis	GO_BiologicalProcess-EBI-UniProt-GOA_10.12.2019_00h00	1,50E-05
GO:003049	maturation of SSU-rRNA	GO_BiologicalProcess-EBI-UniProt-GOA_10.12.2019_00h00	1,44E-04
GO:190236	negative regulation of RNA catabolic process	GO_BiologicalProcess-EBI-UniProt-GOA_10.12.2019_00h00	4,33E-04
GO:004348	RNA stabilization	GO_BiologicalProcess-EBI-UniProt-GOA_10.12.2019_00h00	2,51E-04
GO:190237	negative regulation of mRNA catabolic process	GO_BiologicalProcess-EBI-UniProt-GOA_10.12.2019_00h00	2,51E-04
GO:004825	mRNA stabilization	GO_BiologicalProcess-EBI-UniProt-GOA_10.12.2019_00h00	1,53E-04
GO:004225	ribosome biogenesis	GO_BiologicalProcess-EBI-UniProt-GOA_10.12.2019_00h00	3,35E-14
GO:004227	ribosomal large subunit biogenesis	GO_BiologicalProcess-EBI-UniProt-GOA_10.12.2019_00h00	6,15E-07
GO:000636	rRNA processing	GO_BiologicalProcess-EBI-UniProt-GOA_10.12.2019_00h00	5,04E-10
GO:000047	maturation of LSU-rRNA	GO_BiologicalProcess-EBI-UniProt-GOA_10.12.2019_00h00	8,09E-07
GO:001607	rRNA metabolic process	GO_BiologicalProcess-EBI-UniProt-GOA_10.12.2019_00h00	7,24E-10
GO:009732	response to antineoplastic agent	GO_BiologicalProcess-EBI-UniProt-GOA_10.12.2019_00h00	7,51E-09
GO:000704	cell-cell junction assembly	GO_BiologicalProcess-EBI-UniProt-GOA_10.12.2019_00h00	5,51E-06
GO:007168	response to indole-3-methanol	GO_BiologicalProcess-EBI-UniProt-GOA_10.12.2019_00h00	5,64E-10
GO:009730	cellular response to alcohol	GO_BiologicalProcess-EBI-UniProt-GOA_10.12.2019_00h00	2,19E-06
GO:007168	cellular response to indole-3-methanol	GO_BiologicalProcess-EBI-UniProt-GOA_10.12.2019_00h00	2,42E-10
GO:004329	apical junction assembly	GO_BiologicalProcess-EBI-UniProt-GOA_10.12.2019_00h00	3,19E-04

Supplementary Table 2. Biological Process GO terms of biotinylated-N6L pull-down proteins.

References

1. Bindea, G., Galon, J., Mlecnik, B., 2013. CluePedia Cytoscape plugin: pathway insights using integrated experimental and in silico data. Bioinformatics 29, 661–663. https://doi.org/10.1093/bioinformatics/btt019
2. Bindea, G., Mlecnik, B., Hackl, H., Charoentong, P., Tosolini, M., Kirilovsky, A., Fridman, W.-H., Pagès, F., Trajanoski, Z., Galon, J., 2009. ClueGO: a Cytoscape plug-in to decipher functionally grouped gene ontology and pathway annotation networks. Bioinformatics 25, 1091–1093. https://doi.org/10.1093/bioinformatics/btp101
3. Callebaut, C., Jacotot, E., Guichard, G., Krust, B., Rey-Cuille, M.-A., Cointe, D., Benkirane, N., Blanco, J., Muller, S., Briand, J.-P., Hovanessian, A.G., 1996. Inhibition of HIV Infection by Pseudopeptides Blocking

Viral Envelope Glycoprotein-Mediated Membrane Fusion and Cell Death. Virology 218, 181–192. https://doi.org/10.1006/viro.1996.0178
4. Destouches, D., Page, N., Hamma-Kourbali, Y., Machi, V., Chaloin, O., Frechault, S., Birmpas, C., Katsoris, P., Beyrath, J., Albanese, P., Maurer, M., Carpentier, G., Strub, J.-M., Dorsselaer, A.V., Muller, S., Bagnard, D., Briand, J.P., Courty, J., 2011. A Simple Approach to Cancer Therapy Afforded by Multivalent Pseudopeptides That Target Cell-Surface Nucleoproteins. Cancer Res 71, 3296–3305. https://doi.org/10.1158/0008-5472.CAN-10-3459
5. Doncheva, N.T., Morris, J.H., Gorodkin, J., Jensen, L.J., 2019. Cytoscape StringApp: Network Analysis and Visualization of Proteomics Data. J. Proteome Res. 18, 623–632. https://doi.org/10.1021/acs.jproteome.8b00702
6. Kutmon, M., Riutta, A., Nunes, N., Hanspers, K., Willighagen, E.L., Bohler, A., Mélius, J., Waagmeester, A., Sinha, S.R., Miller, R., Coort, S.L., Cirillo, E., Smeets, B., Evelo, C.T., Pico, A.R., 2016. WikiPathways: capturing the full diversity of pathway knowledge. Nucleic Acids Res. 44, D488-494. https://doi.org/10.1093/nar/gkv1024
7. Shannon, P., Markiel, A., Ozier, O., Baliga, N.S., Wang, J.T., Ramage, D., Amin, N., Schwikowski, B., Ideker, T., 2003. Cytoscape: a software environment for integrated models of biomolecular interaction networks. Genome Res. 13, 2498–2504. https://doi.org/10.1101/gr.1239303
